# Timber-colonizing gram-negative bacteria as potential causative agents of respiratory diseases in woodworkers

**DOI:** 10.1007/s00420-021-01829-1

**Published:** 2022-01-11

**Authors:** Angelina Wójcik-Fatla, Barbara Mackiewicz, Anna Sawczyn-Domańska, Jacek Sroka, Jan Siwiec, Mariola Paściak, Bogumiła Szponar, Krzysztof Pawlik, Jacek Dutkiewicz

**Affiliations:** 1 Department of Health Biohazards and Parasitology, Institute of Rural Health, Jaczewskiego 2, 20-090 Lublin, Poland; 2 Department of Pneumology, Oncology and Allergology, Medical University of Lublin, Lublin, Poland; 3 Department of Immunology of Infectious Diseases, Hirszfeld Institute of Immunology and Experimental Therapy, Polish Academy of Sciences, Wrocław, Poland; 4 Department of Microbiology, Hirszfeld Institute of Immunology and Experimental Therapy, Polish Academy of Sciences, Wrocław, Poland; 5 Department of Parasitology and Invasive Diseases, National Veterinary Research Institute, Puławy, Poland

**Keywords:** Occupational health, Wood dust, Gram-negative bacteria, Hypersensitivity pneumonitis, Endotoxin, Microvesicles, Toxic pneumonitis, *Pantoea agglomerans*, *Rahnella*

## Abstract

**Occurrence:**

Gram-negative bacteria occur commonly in the inner tissues of stored coniferous and deciduous timber, showing a marked variation in numbers. The greatest maximal numbers are found in the sapwood of coniferous timber. The common constituents of the Gram-negative biota are potentially pathogenic species of *Enterobacteriaceae* family of the genera *Rahnella*, *Pantoea*, *Enterobacter,* and *Klebsiella*. The air of wood-processing facilities is polluted with the wood-borne Gram-negative bacteria and produced by them endotoxin, as demonstrated worldwide by numerous studies.

**Effects:**

There are three potential pathways of the pathogenic impact of wood-borne Gram-negative bacteria on exposed woodworkers: allergic, immunotoxic, and infectious. Allergic impact has been underestimated for a long time with relation to Gram-negative bacteria. Hopefully, the recent demonstration of the first documented case of hypersensitivity pneumonitis (HP) in woodworkers caused by *Pantoea agglomerans* which developed in extremely large quantities in birch sapwood, would speed up finding of new wood-related cases of HP caused by Gram-negative bacteria. The second pathway is associated with endotoxin, exerting strong immunotoxic (excessively immunostimulative) action. It has been demonstrated that endotoxin is released into wood dust in the form of nano-sized microvesicles, by peeling off the outer membrane of bacteria. Endotoxin microvesicles are easily inhaled by humans together with dust because of small dimensions and aerodynamic shape. Afterwards, they cause a nonspecific activation of lung macrophages, which release numerous inflammatory mediators causing an inflammatory lung reaction, chest tightness, fever, gas exchange disorders, and bronchospasm, without radiographic changes. The resulting disease is known as “Organic Dust Toxic Syndrome” or “toxic pneumonitis.” The potential third pathway of pathogenic impact is infection. The suspected species is *Klebsiella pneumoniae* that may occur commonly in wood dust; however, until now this pathway has not been confirmed.

**Conclusion:**

Summarizing, Gram-negative bacteria-inhabiting timber should be considered, besides filamentous fungi and actinobacteria, as important risk factors of occupational disease in woodworkers that could be either HP with allergenic background or toxic pneumonitis elicited by endotoxin.

**Supplementary Information:**

The online version contains supplementary material available at 10.1007/s00420-021-01829-1.

## Introduction

Millions of people worldwide are exposed every day to the inhalation of wood dust (IARC 2016). This group includes workers employed at processing of wood in industrial premises (sawmills, plywood and chipboard factories, paper mills, wood pellet production facilities), craftsmen (joiners, carpenters), individuals occasionally exposed to wood (for example at house renovation) and those composting wood or using wood chips as a biofuel or bedding for animals.

Wood dust released into air of breathing zone during industrial processing of timber may contain large amounts of microorganisms developing in bark or in inner wood tissues: sapwood and heartwood. When inhaled by exposed woodworkers, they may initiate pathologic reactions in the lungs, leading to diseases like hypersensitivity pneumonitis (HP, extrinsic allergic alveolitis) or organic dust toxic syndrome (ODTS, toxic pneumonitis) (Eduard et al. 1994; Selman et al. 2012; Nogueira et al. 2019). Filamentous fungi (mostly of the genera *Aspergillus*, *Penicillium* and *Rhizopus*) have been identified as the most common cause of these diseases (NIOSH 1987; Færden et al. 2014; Nogueira et al. 2019), followed by actinobacteria (NIOSH 1987). Occupational diseases in woodworkers could be also elicited by the action of the constituents of wood itself, such as terpenes or plicatic acid causing asthma (Chan-Yeung 1994; Demers et al. 2000) or so far not identified constituents causing nasal adenocarcinoma (IARC 2016; Siew et al. 2017).

For a long time, Gram-negative bacteria have not been identified as causative agents of the occupational respiratory disease in woodworkers despite their abundant occurrence in inner wood tissues (Dutkiewicz et al. 1992a), and the presence of endotoxin in the air of wood industry facilities polluted with wood dust (Wilhelmsson et al. 1984; Asgedom et al. 2020). The situation changed when Mackiewicz et al. (2019) described for the first time the series of HP cases in woodworkers caused by a ubiquitous Gram-negative bacterium *Pantoea agglomerans*. The results of that study prompted us to prepare a review in a broader perspective, summarizing the current knowledge on the occurrence of Gram-negative bacteria in various woods and their potential pathogenic effects.

## Gram-negative bacteria in various woods and wood-processing facilities

### Ecology

Studies conducted in the second half of twentieth century demonstrated the presence of bacteria in wood tissues, both in the central, dead heartwood and in the peripheral live sapwood conducting water and nutrients (Greaves 1971; Rossell et al. 1973; Liese 1975). Their presence was detected in standing (live) trees but rather in low levels (Prażmo and Dutkiewicz 2000). The exception are some pathologic conditions such as “wetwood,” characterized by the presence a water-soaked tissue colonized by bacteria, often Gram negative (*Enterobacter*, *Pantoea*, *Pseudomonas*) in the central core of the heartwood of some deciduous trees (elms, poplars, willows, maples, birches) (Murdoch and Campana 1983; Scott 1984; Prażmo et al. 1996).

Much more bacteria have been found in timber logs stored in lumber yards or in forest. Bacteria, including Gram-negative species, are regarded as early colonizers of stored timber (Greaves 1971; Rossell et al. 1973), preceding the growth of wood-rotting filamentous fungi, causing rapid decay of wood. Bacteria themselves are also able to decompose wood tissues. According to Singh et al. (2016) Gram-negative bacteria can degrade the walls of wood cells by two processes described as “tunneling” and “erosion.”

### Levels of gram-negative bacteria in stored timber, commonest genera, levels of endotoxin

At first, the studies on bacteria in wood were only qualitative, until an introduction of special wood-grinding drills enabled quantitative determination of microorganisms by culture per weight unit of wood. Recently the application of molecular methods increased an accuracy of taxonomic identification of bacteria (Johnston et al. 2016) but had not provided a sufficient solution for reliable quantitative measurements.

Table 1 presents the results of the studies performed in the years 1989–2000 on Gram-negative bacteria colonizing timber logs stored in lumber yards and forests near sawmills in Poland and USA, as potential sources of airborne contamination in wood-processing facilities. Wood samples were taken from transverse sections of the logs with an original, manually operated drilling device [U.S. Patent 5,078,553 assigned Jan. 7, 1992] that collects the pulverized wood into a flask attached beneath the bit in a one-step sterile process (Dutkiewicz et al. 1989). This approach provided a reliable insight into bacterial biota of various woods as a potential respiratory hazard for woodworkers.

**Table 1:** Levels of Gram-negative bacteria and endotoxin in logs of various tree species, stored on lumber yard or in forest

Species of timber log, location	Tissue	Concentration of gram-negative bacteria (CFU × 10^3^ g^−1^, range)	Prevailing genera of gram-negative bacteria	Percent of total microbiota (%, range)^a^	Concentration of endotoxin^b^ (ng × 10^3^ g^−1^, range)	References
Scots pine (*Pinus sylvestris*), Lubelskie,Poland	Sapwood	0.0–6,050,400.0	*Rahnella*, *Enterobacter*	0.0–100	0.0–2000.0	(Dutkiewicz 1989; Prażmo et al. 2000; Prażmo and Dutkiewicz 2000)
	Heartwood	0.0–24.0	*Enterobacter*	0.0–99.8	20.0–40.0	
Norway spruce (*Picea abies*), Małopolska, Poland	Sapwood	0.0–200.0	*Enterobacter, Rahnella, Pantoea*	N. t	N. t	Prażmo and Dutkiewicz (2000)
	Heartwood	0.0–33.0	*Pseudomonas*	N. t	N. t	
Silver fir (*Abies alba*), Małopolska, Poland	Sapwood	0.0–2000.0	*Pantoea*, *Rahnella*, *Enterobacter*	N. t	N. t	Prażmo and Dutkiewicz (2000)
	Heartwood	0.0–160.0	*Rahnella, Pantoea, Pseudomonas*	N. t	N. t	
White warty birch (*Betula pendula*), Lubelskie, Poland	Sapwood	0.0–4.0	*Pantoea*, *Acinetobacter*	0.0–28.6	N. t	(Dutkiewicz 1989; Prażmo et al. 1996; Prażmo and Dutkiewicz 2000)
	Heartwood	0.0–400.0	*Pseudomonas, Rahnella, Pantoea*	N. t	0.0–1710.0	
European hornbeam (*Carpinus betulus*),Lubelskie, Poland	Sapwood	0.0–5.0	*Pantoea*, *Acinetobacter*	0.0–7.9	N. t	Dutkiewicz (1989)
European alder (*Alnus glutinosa*),Lubelskie, Poland	Sapwood	0.0	None	0.0	N. t	Dutkiewicz (1989)
European beech (*Fagus sylvatica*), sapwood,Lubelskie, Poland	Sapwood	0.01–46.0	*Rahnella*, *Pantoea*, *Enterobacter*	N. t	N. t	(Prażmo et al. 2000; Prażmo and Dutkiewicz 2000)
English oak (*Quercus robur*), Lubelskie,Poland	Sapwood	0.0–1.0	*Pantoea*	N. t	N. t	Prażmo and Dutkiewicz (2000)
	Heartwood	0.0	None	N. t	N. t	
American basswood (*Tilia* *americana*), WV,USA	Sapwood	0.0–300.0	*Pantoea*, *Klebsiella*	0.0–39.1	0.04–33.0	Dutkiewicz et al. (1992a)
	Heartwood	0.0–12,000.0	*Pseudomonas*	0.0–99.9	0.13–0.4	
	Bark	0.0–650.0	*Acinetobacter*, *Pantoea*	0.0–20.6	0.22–1.3	
Soft maple (*Acer saccharinum*), WV, USA	Sapwood	0.0–4.5	*Pantoea*	0.0–50.0	0.16–1.0	Dutkiewicz et al. (1992a)
	Heartwood	0.0–6.0	*Pantoea*	0.0–25.0	0.003–0.13	
	Bark	0.0–3.7	*Pseudomonas*	0.0–1.2	0.13–0.65	
Black cherry (*Prunus serotina*), WV,USA	Sapwood	0.0	None	0.0	0.0016–0.13	Dutkiewicz et al. (1992a)
	Heartwood	0.0	None	0.0	0.0011–0.033	
	Bark	0.0–2.5	*Acinetobacter*	0.0–0.1	0.033–0.16	
Black locust (*Robinia pseudoacacia*), WV,USA	Sapwood	0.0–5.5	*Agrobacterium*	0.0–60.0	0.16–0.5	Dutkiewicz et al. (1992a)
	Heartwood	0.0–5.2	*Agrobacterium*	0.0–11.8	0.01–0.5	
	Bark	0.0–7.5	*Pseudomonas*	0.0–97.6	0.0–26.0	
Red oak (*Quercus coccinea*), WV,USA	Sapwood	0.0–3.2	*Pseudomonas*	0.0–1.0	0.013–0.16	Dutkiewicz et al. (1992a)
	Heartwood	0.0	None	0.0	0.002–0.006	
	Bark	0.0	None	0.0	0.0–2.1	
White poplar (*Populus alba*), WV,USA	Sapwood	0.0–7.3	*Pseudomonas*	0.0–99.6	0.007–10.0	Dutkiewicz et al. (1992a)
	Heartwood	0.0–5.3	*Pseudomonas*	0.0–50.0	0.002–1.3	
	Bark	0.0–5.7	*Pseudomonas*	0.0–31.3	0.2–6.5	

*N. t.* not tested, *WV* West Virginia

^a^Estimated by recalculating authors’ data

^b^Assuming 1 ng = 10 EU

The presence of Gram-negative bacteria was detected in the inner tissues of 12 kinds of stored coniferous and deciduous timber logs out of 14 examined. The concentrations of Gram-negative bacteria showed a marked variation, ranging from 0.0 to 6.05 × 10^9^ CFU g^−1^. In general, the greater maximal numbers of bacteria were noted in coniferous than in deciduous timber. In coniferous logs, the maximal concentrations of bacteria in live tissue of sapwood were distinctly higher than in dead tissue of heartwood. By contrast, in deciduous logs, the concentrations of bacteria in the sapwood and heartwood were similar. The numbers in bark (determined only in the samples from USA) were generally similar to those found in the inner tissues. Among the strains isolated from coniferous and deciduous timber in Poland dominated those belonging to family *Enterobacteriaceae* (e.g., *Pantoea spp.*, *Rahnella spp.*, *Enterobacter spp.*), while among those isolated from deciduous timber in USA, the most prevalent were strains belonging to the family *Pseudomonadaceae* (*Pseudomonas* spp.). Gram-negative bacteria formed a marked proportion of the total microbiota (bacteria + fungi) isolated from timber, which varied within wide limits from 0.0 to100% (Table 1).

A part of wood samples taken in Poland and all the samples taken in the USA were examined for the content of endotoxin, a macromolecular constituent of the cell wall of Gram-negative bacteria which may exert respiratory toxicity when inhaled (discussed in detail in the further part of this article). The tested samples contained the biologically active endotoxin in the wide range of 0.0–2000.0 × 10^3^ ng g^−1^ (Table 1). In the samples taken in the USA, a highly significant correlation (*P* < 0.001) was found between the levels of Gram-negative bacteria and endotoxin (Dutkiewicz et al. 1992a). No significant differences could be found between the concentrations of endotoxin in bark versus inner wood tissues (sapwood and heartwood).

### Levels of gram-negative bacteria in the air of wood-processing facilities, commonest genera, levels of endotoxin

Table 2 presents levels of Gram-negative bacteria occurring in the air of breathing zone of various wood-processing facilities contaminated with wood dust, located in Australia, Italy, Poland, and Switzerland. The numbers of bacteria ranged from 0.0 to 21.9 CFU × 10^3^ m^−3^; however, the interpretation of the data was hampered by the lack of widely accepted allowable values for the numbers of airborne microorganisms and their products. In 6 out of 13 cases, the maximal numbers exceeded a value of 1.0 CFU × 10^3^ m^−3^ which was proposed as allowable by Malmros et al. (1992) for premises polluted with organic dusts. Similarly as in timber, two groups of Gram-negatives strains may be distinguished among the strains isolated from the air contaminated with wood dust. The first, slightly more numerous, was formed by the bacteria classified within the *Enterobacteriaceae* family (e.g., *Rahnella* spp., *Pantoea* spp., *Enterobacter* spp., *Klebsiella* spp.) while the second consisted mostly of the members of *Pseudomonadaceae* family (e.g., *Pseudomonas* spp., *Flavimonas* spp., *Burkholderia* spp.).

**Table 2:** Concentration of Gram-negative bacteria in the air of wood-processing facilities

Country	Type of facility	Concentration of Gram-negative bacteria in the air (CFU × 10^3^ m^−3^) (range)	Prevailing genera of gram-negative bacteria	Percent of total microbiota (%)^a^	References
Finland	Paper mill	1.3^b^	*Klebsiella*	N. e	Niemela et al. (1985)
Poland	Sawmill processing birch (*Betula pendula*) logs	0.0–0.48	*Pseudomonas*	N. e	Prażmo et al. (1996)
Australia	Sawmills processing eucalyptus	3.3–14.1	N. r	8.1–44.5	Alwis et al. (1999)
Australia	Joineries (processing various woods)	0.4–15.1	N. r	7.7–41.8	Alwis et al. (1999)
Poland	Sawmill processing pine (*Pinus sylvestris*) logs	0.0–0.6	*Rahnella*, *Enterobacter*, *Pantoea*	N. e	Prażmo et al. (2000)
Poland	Sawmill processing beech (*Fagus sylvatica*) logs	7.8–13.0	*Rahnella*	N. e	Prażmo et al. (2000)
Poland	Sawmills	0.04–21.9	*Rahnella*	0.3–55.0	Dutkiewicz et al. (2001a; b, c)
Poland	Fiberboard and chipboard factories	0.3–5.7	*Acinetobacter*, *Pantoea*, *Pseudomonas*, *Rahnella*	4.0–25.0	Dutkiewicz et al. (2001a; b, c)
Poland	Furniture factories	0.0–5.7	*Rahnella*, *Pseudomonas*, *Acinetobacter*	0.0–20.6	Krysińska-Traczyk et al. (2002)
Poland	Paper mills	0.01–0.31	*Enterobacter*, *Pantoea*, *Rahnella*, *Klebsiella*	N. e	Prażmo et al. (2003)
Switzerland	Sawmills	0.0–0.8	*Burkholderia*, *Flavimonas*, *Pseudomonas*	0.0–2.5	Oppliger et al. (2005)
Italy	Sawmills and carpentries	0.0–0.16	*Klebsiella*, *Pantoea*, *Enterobacter*, *Aeromonas*	0.0–5.5	Gioffré et al. (2012)
Poland	Joineries	≤ 0.08	*Flavimonas*	≤ 1.0	Górny et al. (2017)
Poland	Wood pellet production	0.0	None	0.0	Górny et al. (2019)

*N. e* not estimated, *N. r.* not reported

^a^Estimated by recalculating authors’ data

^b^Upper limit of coliform bacteria

In contrast to the potential allergenic role of Gram-negative bacteria that is largely underestimated, their role as producers of endotoxin causing febrile reaction among woodworkers has been appreciated by many authors engaged in the protection of the health of woodworkers. This was reflected by studies on the levels of airborne endotoxin in wood-processing facilities polluted with wood dust. Table 3 presents the results of the 21 studies performed in 12 countries. As seen, these levels ranged within wide limits between 10^–2^ and 10^3^ ng/m^3^. Maximal values exceeded the allowable exposure level (9 ng/m^3^ ~ 90 EU/m^3^ proposed by the Health Council of the Netherlands, 2010) in 13 out of 21 cases.

**Table 3:** Concentration of bacterial endotoxin in the air of wood-processing facilities

Country	Type of facility	Concentration of endotoxin (ng m^−3^) (average values)	References
Sweden	Furniture factories	1.2–350.0	Wilhelmsson et al. (1984)
Poland	Sawmill	75.0	Prażmo et al. (1996)
Australia	Sawmills	1.3–21.1	Alwis et al. (1999)
Australia	Joineries	0.7–9.8	Alwis et al. (1999)
Sweden	Paper mill (bark cleaning)	23.0–220.0	Rylander et al. (1999)
Canada (British Columbia)	Sawmills	1.0–3.6	Dennekamp et al. (1999)
Canada (Quebec)	Sawmills	32.3–594.4^a^	Duchaine et al. (2000)
New Zealand	Sawmills	2.8–29.5^a^	Douwes et al. (2000)
Poland	Sawmills	240.0–4000.0	Dutkiewicz et al. (2001a)
Poland	Fiberboard and chipboard factories	0.0125–197.4^a^	Dutkiewicz et al. (2001c)
New Zealand	Plywood mill	1.2 –7.6^a^	Fransman et al. (2003)
Poland	Paper mill	20.0–207.7	Prażmo et al. (2003)
Tanzania	Joineries	4.0–38.4^a^	Rongo et al. (2004)
Switzerland	Sawmills	0.04–1.8^a^	Oppliger et al. (2005)
USA	Joineries	1.1	Harper and Andrew (2006)
Croatia	Sawmills	26.2–28.1^a^	Pipinić et al. (2010)
Italy	Sawmills and carpentries	0.14–6.93	Gioffré et al. (2012)
Poland	Joineries	2.51	Górny et al. (2017)
Poland	Wood pellet production	4.1–214.7	Górny et al. (2019)
Ethiopia	Particleboard factories	6.2^a^	Asgedom et al. (2020)
Norway	Sawmills	1.2^a^	Straumfors et al. (2020)

^a^Value(s) converted from Endotoxin Units (EU) assuming 10 EU = 1 ng

## Gram-negative bacteria as a potential cause of respiratory disease in woodworkers

### Allergy

A massive occupational exposure to particulate or liquid bioaerosols may result in hypersensitivity pneumonitis (HP). This is usually an occupational disease of the peripheral lung tissue, which in advanced stage may lead to interstitial fibrosis. The disease is caused by the interaction of immunocompetent cells, classified as Th1 allergic response, with the large quantities of microbial or animal allergens associated with organic dusts. The pathogenic reaction leads to lymphocytic and often granulomatous inflammation of the lower parts of lungs and finally to fibrosis (Selman et al. 2012; Quirce et al. 2016; Sforza and Marinou 2017; Nogueira et al. 2019; Greenberger 2019).

Since 1932, at least 22 papers were published in which HP cases caused by the occupational exposure to the inhalation of wood dust were described. Of these, in 17 filamentous fungi (belonging to *Alternaria*, *Aspergillus*, *Aureobasidium*, *Cryptostroma*, *Graphium*, *Mucor*, *Paecilomyces*, *Penicillium*, *Pullularia*, *Rhizopus*, and *Trichoderma* genera) were determined as a cause of disease, in two thermophilic bacteria (*Thermoactinomyces vulgaris*, *Saccharomonospora viridis*), in one endospore-forming bacillus (*Bacillus subtilis*), and in one wood (Cabreuva) itself (Towey et al. 1932; Minárik et al. 1983; NIOSH 1987; Eduard et al. 1994; Selman et al. 2012; Færden et al. 2014; Nogueira et al. 2019).

To the best of our knowledge, none Gram-negative bacterium was described until recently as a cause of HP in woodworkers or any other individuals exposed to wood dust. In our recent study (Mackiewicz et al. 2019), a Gram-negative bacterium *P. agglomerans* was identified as a main causative agent of acute HP in 5 workers employed as sawyers in the furniture factory (3 females and 2 males, aged 40–53 years), exposed to the inhalation of dust from white warty birch (*Betula pendula*) for 1–4 years. It is noteworthy that all the patients were exposed before to the dust from Scots pine (*Pinus sylvestris*) and had not any adverse symptoms until changing production from pine to birch.

All patients reported work-related symptoms associated with exposure to birch dust. Chest X-ray revealed interstitial diffuse changes mostly in lower and middle lung fields, while in high-resolution computed tomography (HRCT), ground-glass attenuations were observed. Bronchoalveolar lavage (BAL) demonstrated a typical lymphocytic alveolitis. According to the clinical observation and diagnostic tests, four patients were diagnosed with acute HP and one with subacute HP (Mackiewicz et al. 2019).

**Fig. 1:**
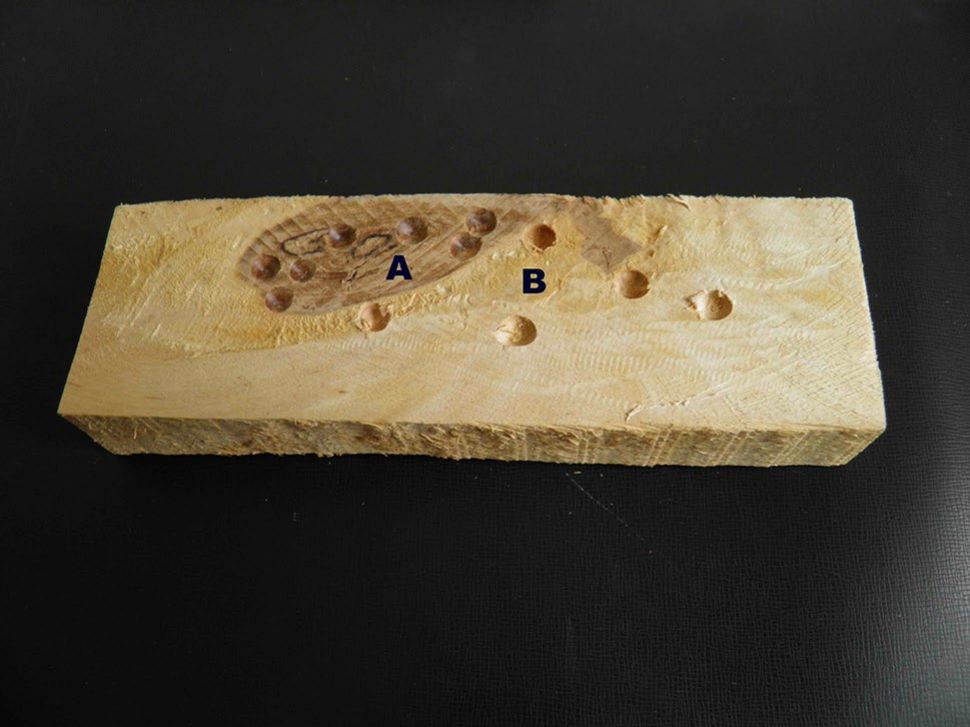
Cylindrical block cut from the transverse section of birch (*Betula pendula*) log. **A** central, brownish ring; **B** peripheral, yellowish ring. Hollows in the wood indicate sites of sampling with the drilling device (according to Mackiewicz et al. 2019)

Microbiological examination of the birch wood samples associated with eliciting respiratory and general symptoms in patients, performed with the use of above-mentioned drilling device (Dutkiewicz et al. 1989), revealed an extremely large, site-depending diversity in the concentration and species composition of the microbiome. The concentration of microorganisms colonizing a brownish ring in the central part of the wooden blocks, corresponding to heartwood and denoted as “A,” was about 10,000 times lower compared to those colonizing the peripheral, yellowish ring corresponding to sapwood and denoted as “B” (4.8 × 10^4^ CFU g^−1^ vs. 4.2 × 10^8^ CFU g^−1^) (Fig. 1). A high prevalence of microorganisms in the peripheral ring “B,” presumably consisted of live tissue, could be explained by a better supplementation with nutrients compared to the central ring “A” consisted of dead tissue. The most common organisms colonizing ring “B” were Gram-negative bacteria identified as belonging to the species *Pantoea agglomerans* (38.7% of the total count) and *Aeromonas* spp. (16.6%) and Gram-positive, coryneform actinobacterium, initially determined as *Microbacterium barkeri* (43.7%).

Taking into account the increasing significance of molecular identification compared to that obtained by culture, we compared in this work quantitative versions of both methods with relation to *Pantoea agglomerans*. The results, which have not been published as yet, are included as an “Online ESM” to this article.

Considering the fact that immunopathologic reaction in HP is initiated by an exposure to large amounts of adverse factors, usually microorganisms, and therefore, the organisms prevailing in the material associated with evoking symptoms are almost surely the disease-causing agents (Towey et al. 1932; Pepys et al. 1963; Eduard et al. 1994; Milanowski et al. 1998; Selman et al. 2012; Nogueira et al. 2019); *Pantoea agglomerans* and *Microbacterium barkeri* were selected as antigens for allergological tests aiming to identify causative agent(s) of the disease observed in the patients. All the patients were examined with the inhalation challenge as a basic test and with the test for inhibition of leukocyte migration (MIF) and agar-gel precipitation test as auxiliary ones. In all tests, lyophilized saline extracts of bacterial mass were used in the doses adjusted to the test (Milanowski et al. 1998; Mackiewicz et al. 2019).

**Fig. 2:**
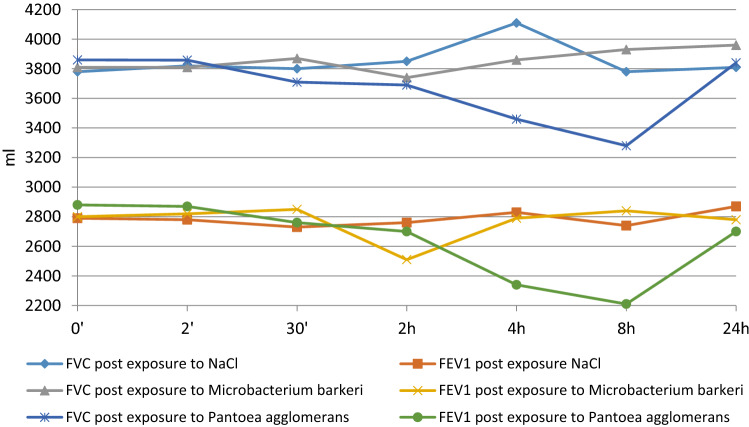
Results of inhalation challenge with the extract of *Pantoea agglomerans* in patient No. 4 compared to the response after exposure to NaCl. Note distinct drops of FVC and FEV_1_ values 8 h post exposure to *P. agglomerans* (unpublished graph)

The inhalation challenge had been used with very low concentrations of bacterial antigens (20 μg ml^−1^) enabling the detection of specific allergy but excluding a possibility of adverse side effects and/or nonspecific reactions caused by endotoxin. The total amount of allergen absorbed by one patient during the test was estimated as 2.16 µg. All five patients responded positively to the challenge with *P. agglomerans* antigen, as assessed by spirometry (drop of the forced vital capacity (FVC) below 10% of the initial value, significant (*P* < 0.01) decrease of FVC and FEV_1_ values, as assessed by *χ*^2^ test) auscultation and symptomatology. The strongest decrease of spirometric values was expressed after 8 h post-challenge (Fig. 2). Two out of five examined patients responded positively to the challenge with *M. barkeri* antigen.

Significant inhibition of leukocyte migration in the presence of the *Pantoea agglomerans* antigen was found in 4 out 5 tested workers. The results indicate that this antigen had a potential to initiate the immunopathologic cellular reaction that is most significant in the pathogenesis of HP. Only in one serum out of five tested workers sera, the presence of precipitin antibodies directed against *P. agglomerans* antigen was revealed suggesting that the time of workers’ exposure was not long enough to produce precipitins that are known markers of long-lasting exposure to adverse antigens, but not necessarily of disease (Pereira et al. 2016; Nogueira et al. 2019). Two out of five patients were positive in the MIF test with *M. barkeri* and none in the precipitin test with this antigen.

In conclusion, *Pantoea agglomerans* was recognized as a primary agent causing HP in the workers exposed to dust from birch wood, and *Microbacterium barkeri* as a secondary agent, probably potentiating the effects of sensitization to Gram-negative bacterium. As preceding microbiological examination of birch wood has not demonstrated large quantities of Gram-negative bacteria (Table 1), the extremely high level of these bacteria found in this work was presumably not associated with the tree species but rather with not yet recognized nutritional and/or microclimatic conditions allowing their expansion in wood tissue. This view can be further supported by an observation that the patients worked for a long time safely with pine wood, which could contain large quantities of Gram-negative bacteria and endotoxin as demonstrated by our earlier studies (Table 1). Therefore, the respiratory hazard should be considered as a combination of nutritional and microclimatic factors that may cause development of the potentially pathogenic bacteria in any kind of timber.

*Pantoea agglomerans* (synonyms: *Enterobacter agglomerans, Erwinia herbicola*), member of the *Enterobacteriaceae* family, recently proposed to be included into *Erwiniaceae*, is probably one of the most common microorganisms living on Earth. Originally plant inhabitant, developing immensely as epi- or endophytic organism in multitude of plant species, it was found also in animal and human tissues, soil, water, and sewage (Dutkiewicz et al. 2015). *P. agglomerans* has been isolated from grain and grain dust in USA and Poland, in the concentrations ranging from 10^5^ to10^7^ CFU g^−1^ (DeLucca et al. 1984; Milanowski et al. 1998; Dutkiewicz et al. 2016a). It has been identified as a common cause of HP in the Polish agricultural workers exposed to the inhalation of organic dusts, mostly from grain (Kuś , 1980a; b; Milanowski et al. 1998), less often from flour (Kuś , 1980a; b), clover (Milanowski et al. 1998), and herbs (Mackiewicz et al. 1999). The bacterium caused also a HP case described in a German farmer (Sennekamp et al. 2012).

The results of the above presented paper by Mackiewicz et al. (2019) clearly demonstrate that *P. agglomerans* could be also an important cause of HP among workers of wood industry.

Little is known about the role of other Gram-negative bacteria present in wood dust as potential causative agents of respiratory disease in exposed individuals. Out of bacteria belonging to *Enterobacteriaceae* family, the primary candidates are genera *Rahnella* and *Enterobacter*. It has been demonstrated by the intradermal skin test that the workers of the sawmill processing coniferous wood (pine), exposed during their work to the inhalation of large quantities of *Rahnella* spp. particles, revealed a high rate (65%) of the early allergic grade 2 reactions of the diameter ≥ 10 mm to the extract of this bacterium, compared to less exposed workers of the sawmill processing deciduous wood (oak and birch) who reacted with the rate of 28.9%, and to members of not exposed reference group who reacted with the rate of 18.7%. These differences proved to be highly significant (*P* < 0.001) (Dutkiewicz et al. 2001b). Unexpectedly, also less exposed workers of furniture factory showed a high rate of positive skin grade 2 reactions, significantly greater compared to the reference group (64.6% vs. 18.7%, *P* < 0.001) (Skórska et al. 2002). Nevertheless, it must be stressed that these data provided only information on immunological, but not clinical sensitization and so far proved only an allergenic potential of *Rahnella*, but not ability to cause bronchial asthma or any other disease.

The allergenic potential may reveal also bacteria of family *Pseudomonadaceae*, especially *Pseudomonas* spp. commonly detected in wood tissue (Table 1) and in air contaminated with wood dust (Table 2). Bernstein et al. (1995) demonstrated that bacteria belonging to this genus may cause HP in the workers exposed to contaminated metalworking fluid aerosols. The furniture factory workers showed a high incidence of precipitin reactions to *Pseudomonas maltophilia*, significantly greater than in reference group (27.1% vs. 0.0%, *P* < 0.01) (Skórska et al. 2002). Although this was only an immunological reactiveness, it should be considered as an important indication for further studies on *Pseudomonas* spp. as a potential cause of allergic diseases resulting from the exposure to wood dust.

## Endotoxin effects

When inhaled during work together with wood dust or other organic dusts, Gram-negative bacteria may affect human health mainly by two pathogenic reactions resulting in lung inflammation. Of which, the first is T-cell-dependent specific allergic reaction leading to hypersensitivity pneumonitis (HP) with radiographic changes, manifested in chronic stage of disease as fibrosis, whereas the second is an unspecific fever reaction without radiographic changes caused by endotoxin, a high-molecular compound produced by Gram-negative bacteria. The type of the reaction depends on both the individual’s characteristics (age, genetic determinants, immunological profile etc.) and circumstances of exposure. Out of Gram-negative bacteria associated with wood dust a special attention should be paid in this respect to the ubiquitous species *Pantoea agglomerans*, recently indicated as an agent causing exacerbations of the chronic obstructive pulmonary disease (COPD) (Shrestha et al. 2021). Earlier, it has been demonstrated experimentally that this species produces as well a potent endotoxin (Dutkiewicz 1976; Rylander and Lundholm 1978).

Endotoxin is a biologically active lipopolysaccharide (LPS) that is an integral component of the outer membrane of the cell wall of Gram-negative bacteria. This is a macromolecular polymer, consisting of hydrophobic (lipidic) and hydrophilic (polysaccharide) moieties. The biological properties of LPS strongly depend on chemical composition and structure. The molecule contains three structural parts: lipid A, core oligosaccharide, and O-specific polysaccharide. Lipid A is responsible for nonspecific pathophysiological responses, including fever, intravascular coagulation, hypotension, or shock. To date, the lipid A part of *P. agglomerans* LPS has conveyed the special attention since it has been shown to contain at least two types of lipid A of different acylation levels (Tsukioka et al. 1997): the hexaacyl lipid A that shows a high biological activity, including endogenous tumor necrosis factor alpha (TNFα) induction, while the activity of the heptaacyl lipid A is slightly lower (Zdorovenko et al. 2017). Specific features of the LPS, the number of fatty acids in lipid A, as well as their distribution, chain length, and stereochemistry, are significant for many activities, including toxicity and pyrogenicity (Zdorovenko et al. 2017, 2018, 2019). To date, biological activity of endotoxins was found to be stronger in the dust-borne *Enterobacteriaceae* species (*Pantoea agglomerans, Rahnella* spp.) than in non-*Enterobacteriaceae* ones (*Acinetobacter calcoaceticus*, *Alcaligenes faecalis*) (Dutkiewicz et al. 1988). Thus, besides *P. agglomerans*, also the strains of *Rahnella*, *Enterobacter* and *Klebsiella* commonly occurring in wood dust (Prażmo et al. 2000; Gioffré et al. 2012) are regarded as producers of a potent, active endotoxin.

**Fig. 3:**
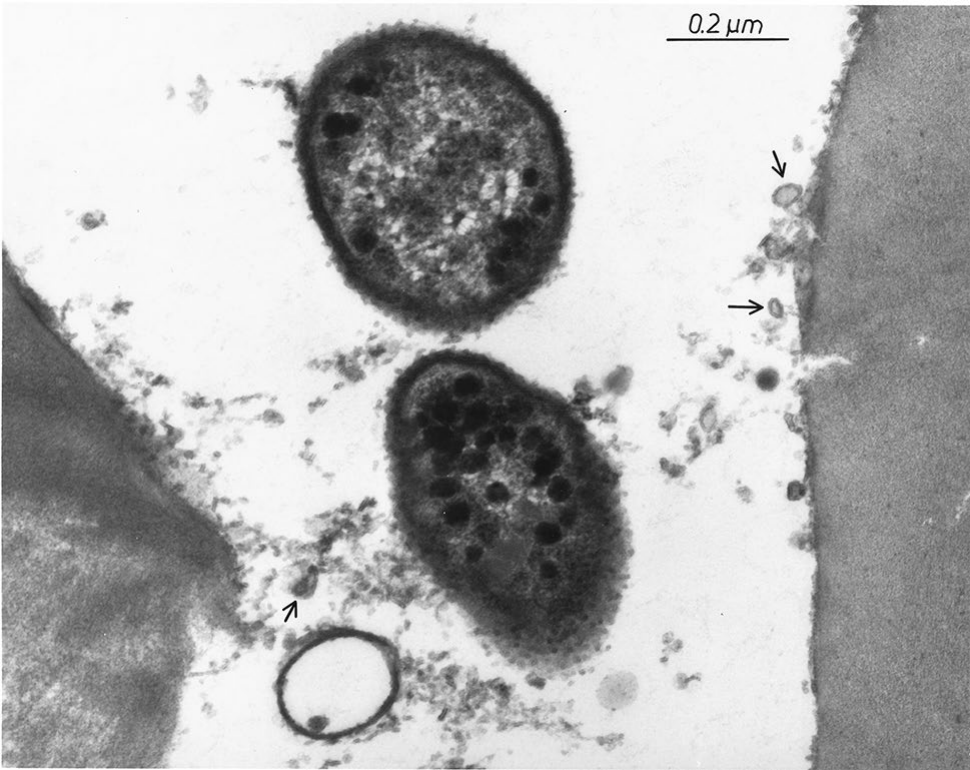
Thin-sectioned sample of pulverized wood from American basswood stained with uranyl acetate and lead citrate, showing two cells of Gram-negative bacteria (presumably *Pantoea agglomerans*) and numerous membrane vesicles (some marked with arrows) in the lumen of a wood cell, TEM (according to Dutkiewicz et al. 1992b)

**Fig. 4:**
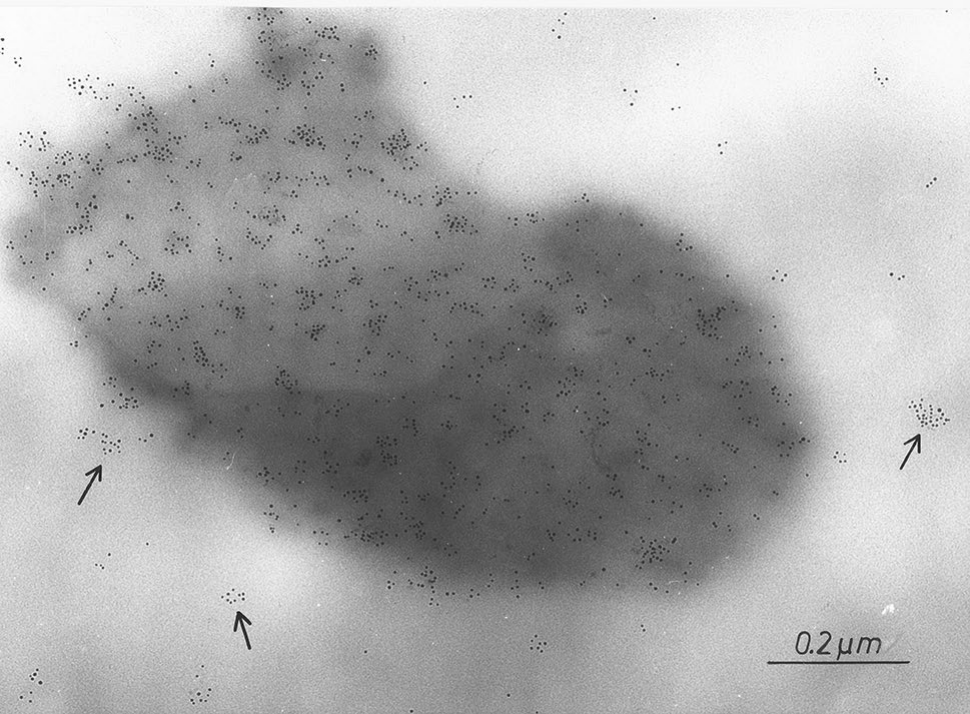
Thin-sectioned sample of pulverized wood from American basswood immunostained with the rabbit antiserum against LPS of *P. agglomerans* and gold-labeled with anti-rabbit IgG. Structure corresponding to the cell of *P. agglomerans* is seen, stained positively with immunogold. Arrows outside the cell show aggregations of gold particles to smaller structures corresponding in shape and size to membrane vesicles, TEM (according to Dutkiewicz et al. 1992b)

**Fig. 5:**
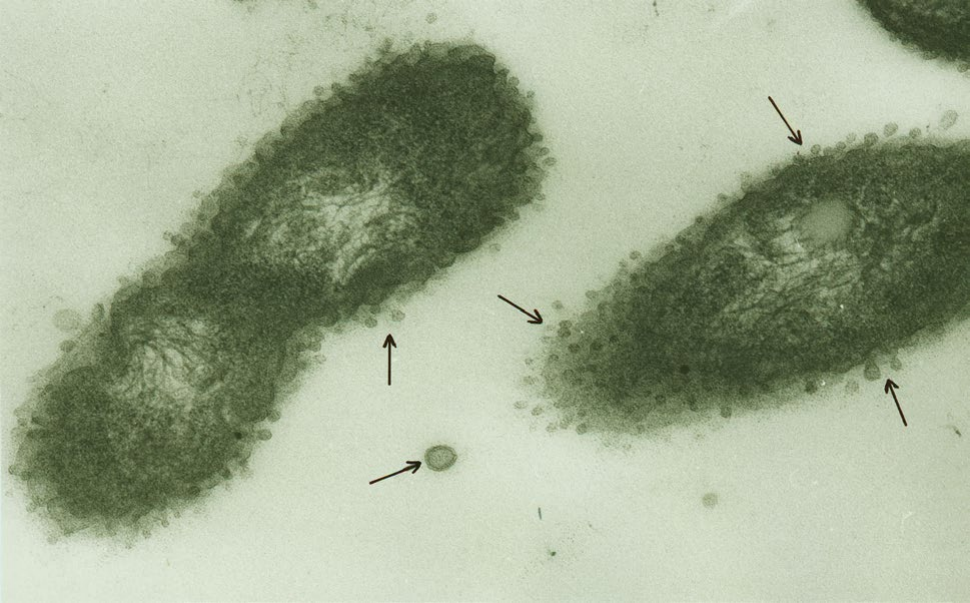
Thin-sectioned sample of pulverized wetwood from white warty birch stained with uranyl acetate and lead citrate, showing two cells of Gram-negative bacteria (presumably *Pseudomonas* spp.) and numerous membrane vesicles (some marked with arrows) peeling from the outer membranes of these bacteria, TEM (according to Prażmo et al. 1996)

Fragmentation of the LPS-containing cell wall, usually following desiccation or fission, easily releases endotoxin-containing particles into dust in the form of microvesicles surrounded by a lipid bilayer and measuring mostly 10–60 nm. These nanoparticles, structurally macromolecular heteropolymers of LPS with proteins and phospholipids of the cell wall, are known in the literature under various names, such as outer membrane vesicles—OMVs (Chudzik and Paściak 2020), extracellular vesicles—EVs (Kim et al. 2013), or ultrafine particles—UFP (Yang et al. 2020). For the first time, environmental release of such nanoparticles has been demonstrated in the sapwood tissue of American basswood (*Tilia americana*) colonized by *Pantoea agglomerans* (Dutkiewicz et al. 1992b). They were released by peeling off the outer membrane of bacterium and enlarged after detachment (Fig. 3). The identity of these microvesicles with parent organism was confirmed by immunostaining with the rabbit antiserum against LPS of *P. agglomerans* followed by labeling with immunogold conjugated with anti-rabbit IgG (Fig. 4). Similarly to biological activity, the ability to release endotoxin particles in the form of microvesicles is strongest among *Enterobacteriaceae* strains. Nevertheless, it has been observed also in other Gram-negative bacteria, for example in *Pseudomonas* species colonizing pathologically changed heartwood tissue of birch (*Betula pendula*) described as “wetwood” (Fig. 5) (Prażmo et al. 1996).

Endotoxin-containing microvesicles (ECMVs) are easily inhaled by humans together with dust because of small dimensions and aerodynamic shape. Afterwards, they cause a nonspecific activation of lung macrophages, which release numerous substances known as inflammatory mediators. Symptoms include an inflammatory lung reaction, chest tightness, fever, gas exchange disorders, and bronchospasm, without radiographic changes (Dutkiewicz et al. 2016a). Immunotoxic disorders caused by the endotoxin inhalation occur mostly in young people and are known under the names “organic dust toxic syndrome” (ODTS) or “toxic pneumonitis.” In earlier studies, cases of this disease most probably caused by *P. agglomerans* endotoxin present in grain or cotton were described, respectively, as “grain fever” (Dutkiewicz et al. 2016a) or “Monday fever” (byssinosis) (Rylander 1981).

Strong immunostimulating properties of ECMVs fraction separated from *Rahnella* sp. or *Pantoea agglomerans* cells by differential sucrose gradients was demonstrated by long-term inhalation experiments in rabbits (Skórska et al. 1996; Dutkiewicz et al. 2005). The experiments showed that microvesicular fraction of endotoxin, corresponding to native form of this compound naturally occurring in the air polluted with wood dust and inhaled by exposed workers, caused a large and highly significant increase in the concentration of the circulating proinflammatory cytokines: total interferon (IFN), interleukin-1α (IL-1α), and tumor necrosis factor α (TNFα). Besides the nonspecific proinflammatory activity, ECMVs fraction elicited also specific cellular and humoral response in exposed rabbits, thus, strengthening the adverse immune stimulation. The results of this experiment suggest that in real life could be only a thin line between the specific allergic reaction(s) causing HP and nonspecific endotoxin reaction(s) causing ODTS, which may even potentiate each other. The airborne, microvesicular endotoxin produced by Gram-negative bacteria probably elicits toxic pneumonitis by itself or may augment the allergenic effects of these bacteria and exacerbate the clinical course of HP.

Immunostimulative properties of dust-borne bacterial microvesicles have been confirmed by Kim et al. (2013) who demonstrated on mouse model that repeated intranasal application of ECMVs fraction separated from mattress dust is able to induce proinflammatory cytokines and neutrophilic pulmonary inflammation. More recently, Yang et al. (2020) raised an interesting hypothesis that the inhalation exposure to low levels of LPS-containing microvesicles may cause Th2-mediated eosinophilic asthma, whereas the exposure to high levels may cause Th17-mediated chronic pulmonary diseases such as neutrophilic asthma, COPD, and lung cancer. Accordingly, these authors regard microvesicles produced by Gram-negative bacteria as a key etiological agent of the chronic pulmonary inflammatory diseases. In the recent review on bacterial extracellular vesicles, Chudzik and Paściak (2020) present a wide range of their vital functions including pathogenic modulation of host’s immune system, mediation in the communication between bacterial cells with the use of signal molecules, horizontal gene transfer, protection of parent cell(s), enzyme-based acquisition of nutrients, and ability of the biofilm formation.

So far, the occupational allergic diseases caused by the inhalation of wood dust were related mainly to filamentous fungi, actinobacteria, and wood constituents; hence, the Gram-negative bacteria in this role are “new players.” In contrast, the importance of bacterial endotoxin as a possible cause of ODTS among woodworkers, along with substances produced by filamentous fungi, such as macromolecular (1 → 3)-β-d-glucan or low-molecular volatile organic compounds (VOCs) is widely considered, which is reflected by the numerous studies worldwide presenting levels of airborne endotoxin in wood-processing facilities (see Table 3). This is an important assessment and indicator of occupational health risk and/or overall work hygiene in examined facilities.

The potential hazard of exposure to endotoxin was demonstrated by Rylander et al. (1999) in Sweden and in Australia by Mandryk et al. (2000) who reported a significant relationship between the levels of endotoxin in airborne wood dust and occurrence of work-related respiratory symptoms in exposed workers. Górny et al. (2019) found a significant correlation between the levels of endotoxin in the air of wood pellet production facilities in Poland, and levels of cytokines known as proinflammatory mediators in the nasal lavage from exposed workers.

To date, descriptions of the individual ODTS cases caused by the inhalation exposure to endotoxin in wood dust are rare. Most probably, one of them is that reported by Weber et al. (1993). These authors described the case of 52 year old male who developed fever, myalgia, and marked dyspnea 12 h after shoveling composted wood chips and leaves. Numerous fungal spores and large endotoxin concentrations ranging from 63.6 to 1.630.0 ng/m^3^ were found in respirable dust samples indicating these factors as probable causative agents of the disease.

## Infection

Little is known about work-related infections caused by Gram-negative bacteria in woodworkers. The risk of pulmonary infection could be associated with the presence of the species *Klebsiella pneumoniae* that has been isolated from wood tissue (Bagley et al. 1978), paper mill effluents (Caplenas et al. 1981), as well as from the air of paper mill and nasal cavities of exposed workers (Niemelä et al. 1985). More recently, this species has been identified, together with *Klebsiella rhinoscleromatis*, by Neghab et al. (2018) as one of the predominant Gram-negative bacteria in the air of sawmills in Iran. All these data are in accordance with the opinion expressed by Munoz et al. (2006) that wood-based bedding is a main source of *Klebsiella pneumoniae* on dairy farms. Nevertheless, to the best of our knowledge, none case of pneumonia caused by this bacterium was described in a person exposed to wood dust.

Out of other timber-colonizing Gram-negative bacteria, *Pantoea agglomerans* and *Rahnella aquatilis* were identified as causative agents of opportunistic infections in humans. The infections occur mostly in immunocompromised persons and are often hospital acquired (Chang et al. 1999; Dutkiewicz et al. 2016b). Moreover, *P. agglomerans* causes wound infections, following piercing or laceration of skin with plant material, such as thorn or wooden splinter. The latter pathway should be considered as a possible pathogen transmission to woodworkers (Dutkiewicz et al. 2016b).

## Epidemiology and clinical diagnosis

Compared to filamentous fungi, the role of Gram-negative bacteria in causing occupational disease in woodworkers is less known, and at present, there are no reliable epidemiological data on this subject. Recently, organic dust toxic syndrome (ODTS, toxic pneumonitis) is probably the most common disorder caused by Gram-negative bacteria and endotoxin present in the airborne wood dust. A significant correlation between the levels of endotoxin and/or Gram-negative bacteria in airborne wood dust and occurrence of work-related symptoms corresponding to ODTS were reported for the populations of workers employed in the Swedish paper mills at debarking the logs (Rylander et al. 1999), as well as for those working in the ‘green sawmills’ (processing fresh logs, mainly eucalypts) in Australia (Mandryk et al. 2000) and in the Iranian sawmills (Neghab et al. 2018). The incidence of ODTS in woodworkers is most probably distinctly higher compared to hypersensitivity pneumonitis (HP). Based on the epidemiological data reported by Malmberg et al. (1988) for Swedish farmers, we may presume for a population of woodworkers a yearly incidence of HP circa 2–3/10,000 workers and for ODTS circa 1/100 workers, i.e., 30–50 times higher. Most probably, these figures tend to decrease along with the technical improvement of ventilation systems in wood-processing facilities.

Flu-like symptoms (dyspnea, cough, fever, shivering) which are characteristic for ODTS, are similar to those appearing in the acute stage of HP. The correct diagnosis of the latter is based on the presence of radiographic changes and specific immune reactions with bacterial antigens, which are usually absent in ODTS. Also, the ODTS symptoms may occur after single massive exposure, whereas those of acute HP appear after repeated exposures needed for the formation of specific immunity (Malmberg et al. 1988).

So far, there are only few information on infectious diseases caused by timber-colonizing Gram-negative bacteria. Despite the presence of potentially infectious species (*Klebsiella pneumoniae*, *Klebsiella rhinoscleromatis*, *Pantoea agglomerans*) in wood and wood dust, the probability of disease in immunocompetent workers is rather low. Such possibility could be considered in the case immunocompromised persons exposed to wood dust containing the above-mentioned pathogens. Diagnosis should be based on the isolation of pathogen from body fluids and identification with biochemical, molecular, and immunological methods.

## Conclusions

1. Gram-negative bacteria occur commonly in the inner tissues of stored coniferous and deciduous timber, showing a marked variation in the numbers. The greatest maximal numbers could be found in the live tissue of sapwood of coniferous timber. The common constituents of the Gram-negative biota are potentially pathogenic species of *Enterobacteriaceae* family of the genera *Rahnella*, *Pantoea*, *Enterobacter,* and *Klebsiella*.
2. The air of the wood-processing facilities is polluted with the wood-borne Gram-negative bacteria and their endotoxin (lipopolysaccharide, LPS), as demonstrated by numerous studies.
3. There are three potential pathways of the pathogenic impact of wood-borne Gram-negative bacteria on exposed woodworkers: allergic, immunotoxic, and infectious. Allergic impact of Gram-negative bacteria has been underestimated for a long time and might contributed to a long delay in the diagnosis of the first case of hypersensitivity pneumonitis (HP) caused by these bacteria. Hopefully, the recent demonstration of the first documented case of HP in woodworkers caused by *Pantoea agglomerans* developing extremely large quantities in birch sapwood would speed up finding of new wood-related cases of HP caused by this species and other Gram-negative bacteria, based on state-of-the-art clinical, immunological, and microbiological methods.
4. The second pathway of the pathogenic impact of wood-borne Gram-negative bacteria is associated with endotoxin, exerting strong immunotoxic (excessively immunostimulative) action. It has been demonstrated that endotoxin is released into wood dust in the form of microvesicles measuring mostly 10–60 nm, by peeling off the outer membrane of bacterium. Endotoxin-containing microvesicles are easily inhaled by humans together with dust because of small dimensions and aerodynamic shape. Afterwards, they cause a nonspecific activation of lung macrophages, which release inflammatory mediators causing an inflammatory lung reaction, chest tightness, fever, gas exchange disorders, and bronchospasm, without radiographic changes. The resulting disease is known as “organic dust toxic syndrome” (ODTS) or “toxic pneumonitis.”
5. The potential third pathway of the pathogenic impact is infection. The suspected species is *Klebsiella pneumoniae* that may occur in wood dust; however, this pathway has not been confirmed.
6. Summarizing, Gram-negative bacteria-inhabiting wood should be considered, together with filamentous fungi and actinobacteria, as important risk factors of occupational disease in woodworkers that could be either HP with allergenic background or toxic pneumonitis elicited by endotoxin.

## Supplementary Information

Below is the link to the electronic supplementary material.

Supplementary file1 (DOCX 27 KB)

## Data Availability

The *Pantoea agglomerans* strain isolated from the birch wood was deposited at the Polish Collection of Microorganisms in the Institute of Immunology and Experimental Therapy of the Polish Academy of Science in Wrocław (Poland) under the number PCM 3041. The sequence of the 16S rRNA gene fragment determined in this study was deposited in the GenBank under accession number MW647906.1 (*Pantoea agglomerans* strain PCM3041 16S ribosomal RNA gene, partial sequence).
