# Supplementary material for: Timber-colonizing gram-negative bacteria as potential causative agents of respiratory diseases in woodworkers
Source: Int Arch Occup Environ Health. 2022 Jan 11;95(6):1179–93. doi: 10.1007/s00420-021-01829-1 (PMC9273545; doi:10.1007/s00420-021-01829-1)

**Annex**

**Microbiological and molecular identification of *Pantoea agglomerans* in wood samples**

**Sample preparations**

Two 5 g samples of the pulverized birch wood were homogenized with a BagMixer 400 SW homogenizer (Interscience, France) for 4 min in 45 ml of a Ringer’s solution (Merck KGaA, Germany). One sample of the homogenate was used for cultures, while the second one was filtered and intended for DNA extraction directly from the wood sample.

**Culture and biochemical tests**

The Columbia blood agar (GRASO Biotech, Poland) was used for *P. agglomerans* subculture with 48 hrs incubation time at 37^o^C. For preparing of dilution series from the cultures (from 1 colony to 10^-6^ CFU ml^-1^), the inoculated tryptic soya agar (BTL, Poland) plates were incubated for 24 hrs at 30^o^C. The density of the suspension corresponded to 1 colony of *P. agglomerans* was determined based on the agar plate 10^-5^ dilution (Fig.6e) and was equal to 58 × 10^6^ CFU ml^-1^. The identification of *P. agglomerans* strain was confirmed by biochemical methods using ENTEROtest 24N (Erba Lachema, Czech Republic) and BIOLOG system (Biolog, Inc., USA). The *Pantoea agglomerans* strain isolated from the birch wood was deposited at the Polish Collection of Microorganisms in the Institute of Immunology and Experimental Therapy of the Polish Academy of Science in Wrocław (Poland) under the number PCM 3041.

**DNA isolation**

Total DNA was isolated from the culture of *P. agglomerans* on Columbia agar with blood using Qiamp DNA Mini Kit (Qiagen, USA) according to the manufacturer’s protocol for Gram-negative bacteria. To plot the slop of the real-time PCR (RT PCR) standard curve, DNA was extracted from one colony of *P. agglomerans* on tryptic soya agar, followed by 10-fold dilutions of DNA in nuclease free water. DNA was also isolated directly from 10-fold dilutions of one colony in physiological solution (up to 10^-6^) used to determine the concentration of *P. agglomerans* by dilution plating (Fig. 8). DNA isolation directly from the birch wood was performed from 5 g sample of the homogenized pulverized wood, which was filtered and used for isolation by Qiamp DNA Mini Kit.

**PCR amplification**

*Pantoea agglomerans* DNA was detected by amplification of 16S rRNA gene fragment using the universal oligonucleotide primers p27f and p1525r according to method by Chun and Goodfellow (1995). Each reaction of 50 µl volume consisted of 1.5 U Taq DNA Polymerase (Qiagen, Germany), 5 µl each of 10 × PCR buffer containing 15 mM MgCl_2_ (Qiagen, Germany) and 2 mM dNTPs (final concentration 0.2 mM; Thermo Scientific, USA), 2 µl 10 µM each primer (Institute of Biochemistry and Biophysics, Poland), 5 µl DNA template and nuclease-free water (Qiagen, Germany). The reaction was performed on C1000 Thermal Cycler (BioRad, USA) under the conditions described by Chun and Goodfellow (1995). Products of amplification were identified in 2% agarose gel (Prona, Basica LE), after electrophoresis in standard conditions and staining with ethidium bromide solution (2 μg/ml). As seen on Figure 7 the positive results were obtained by classic PCR in the case of *P. agglomerans* isolation (from 1 colony) both from Columbia agar with blood (lines 1-2) and tryptic soya agar (lines 4 and 14). Among 5 samples isolated directly from the birch wood, a positive result was obtained in 4 cases (lines 9-13, responding to the results on Fig. 8B). The dilutions series (from 1 colony to 10^-4^ CFU ml^-1^) revealed positive results up to 10^-3^ dilution, both for DNA extracted from dilutions prepared from 1 colony (lines 4-8, responding to the results on Fig. 8A) and for DNA isolates made for each dilution separately (lines 14-18, responding to the results on Fig. 8C), however the last visible amplification product is very weak (lines 7 and 17).

**Sequencing**

DNA sequencing of all positive samples was performed with ABI PRISM 310 Genetic Analyzer (AppliedBiosystems, Inc., Foster City, CA, USA) using Abi Prism Big Dye Terminator v. 3.1. Cycle

Sequencing Kits and Big Dye XTerminator Purification Kit (Applied Biosystems). The results were compared with sequences stored in GenBank database using the BLAST software at the National Center for Biotechnology Information (Bethesda, Maryland, USA). The sequence of the 16S rRNA gene fragment determined in this study was deposited in the GenBank under accession number MW647906.1 (*Pantoea agglomerans* strain PCM3041 16S ribosomal RNA gene, partial sequence).

**Real-time PCR**

Reaction was conducted according to the own method with the use of the same set of primers as in the classic PCR reaction. Each reaction (30 µl) consisted of 15 µl 2× iQ^TM^ SYBR^®^ Green Supermix (Bio-Rad, USA), 0.5 µl 10 µM each of primer, 2 µl DNA template and 12 µl nuclease-free water. The amplification was carried out in Step One Real-Time PCR System (Applied Biosystems, Inc., Foster City, CA, USA) under the following conditions: preincubation at 95°C for 10 min and 40 cycles, each of 15 sec at 95° C, 1 min at 55°C, and 1 min at 72°C. The melt curve protocol was increments between 60°C to 95°C. The standard curve was made from 5 points responding to DNA sample from 1 colony obtained from culture on tryptic soya agar and its dilutions in nuclease free water from 10^-1^ to 10^-4^. Limitation of the methods was the lack of the sample with the known copies number of 16S rRNA gene fragment for using as a positive control in the test. Total DNA isolated from 1 colony has been diluted in nuclease free water (from 10^-1^ to 10^-4^) to prepare standard curve (Fig. 8A). The Ct (cycle threshold) value of samples prepared directly from birch wood (Ct:25-27) corresponded to the Ct of the samples in 10^-3^ dilution (Ct:26) (Fig. 8B). The Ct values of samples isolated directly from dilutions in physiological saline (0.9% sodium chloride) prepared for culture (Fig. 6), were a little higher but comparable with the Ct of samples used in standard curve (Fig. 8C) (for 10^-1^ : 18 vs. 14; for 10^-2^ : 25 vs. 19; for 10^-3^ : 29 vs. 26 and for 10^-4^ :37 vs. 31). The higher Ct values may be the effect of lower efficiency of DNA isolation from bacteria resuspended in physiological saline. In both methods PCR and real-time PCR, limit of *P. agglomerans* detection was for 10^-3^ dilution.

**Reference**:

Chun J, Goodfellow M (1995) A phylogenetic analysis of the genus *Nocardia* with 16S rRNA gene sequences. Int J Syst Bacteriol 45(2):240-245. https://doi.org/10.1099/00207713-45-2-240

**Figure Captions**

**Fig. 6** *Pantoea agglomerans* culture on tryptic soya agar; a: dilution 10^-1^; b: dilution 10^-2^; c: dilution 10^-3^; d: dilution 10^-4^; e: dilution 10^-5^; f: dilution 10^-6^ (Wójcik-Fatla, unpublished materials)

**Fig. 7** Agarose gel electrophoresis of *Pantoea agglomerans* PCR-amplified products. Marker: GeneRuler 1 kb DNA Ladder (Thermo Scientific, USA). Lines 1,2: *P. agglomerans* strain from Columbia blood agar (Accession No.:MW647906.1); line 3: negative control (nuclease-free water); lines 4-8: *P. agglomerans* dilution series from 1 colony DNA isolate to 10^-4^ CFU/ml (in nuclease free water); lines 9-13: *P. agglomerans* isolates directly from the birch wood; lines 14-18: *P. agglomerans* isolated directly from dilutions in physiological solution (from 1 colony to 10^-4^ CFU/ml). Amplification products size: approx. 1500 bp (Wójcik-Fatla, unpublished materials)

**Fig. 8** Real-time PCR standards curves, amplification curves and melting curves of *Pantoea agglomerans* isolates. A: Samples used for standard curve; B: samples isolated directly from birch wood; C: samples isolated from dilutions in physiological solution (Wójcik-Fatla, unpublished materials)


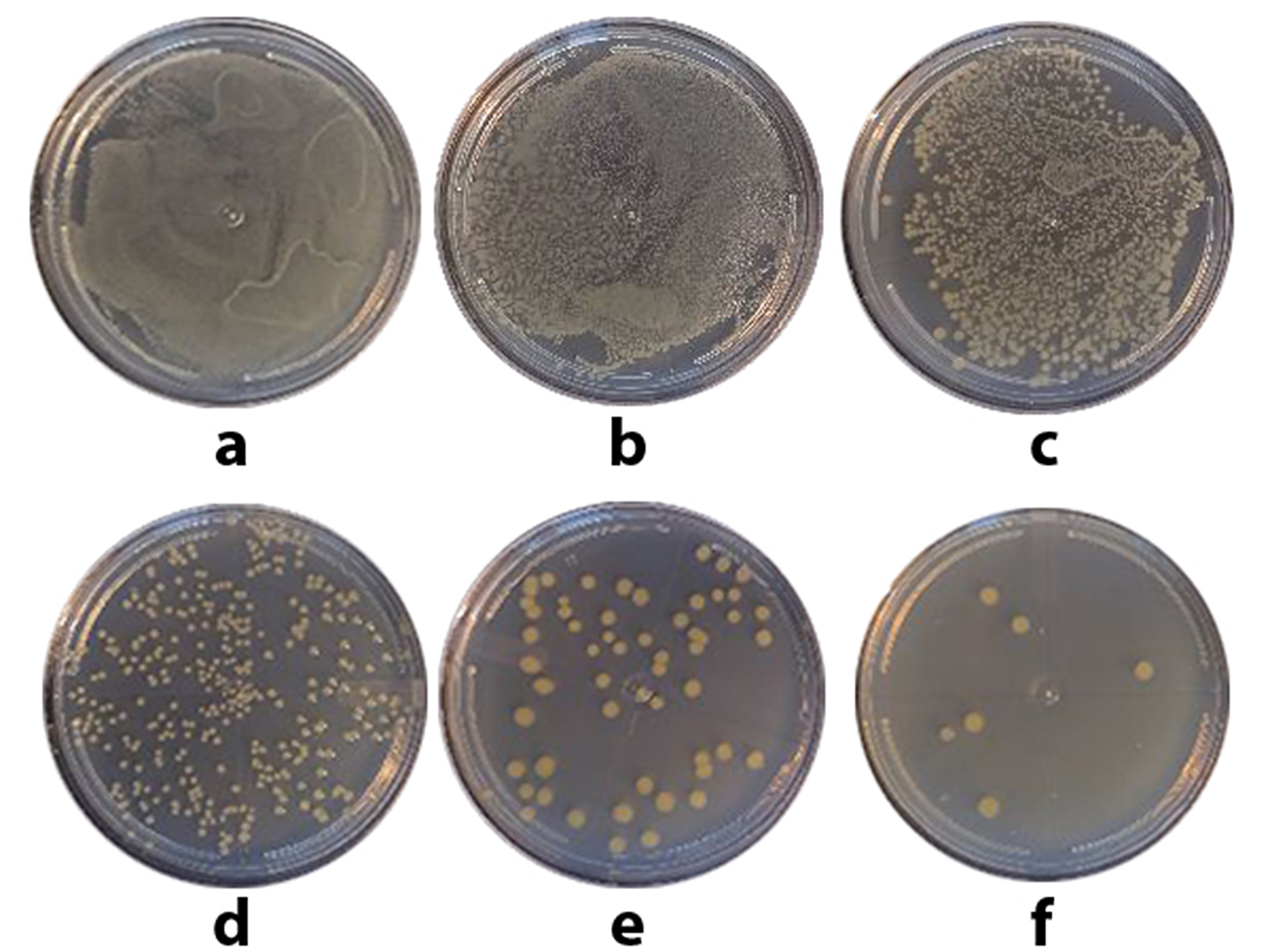

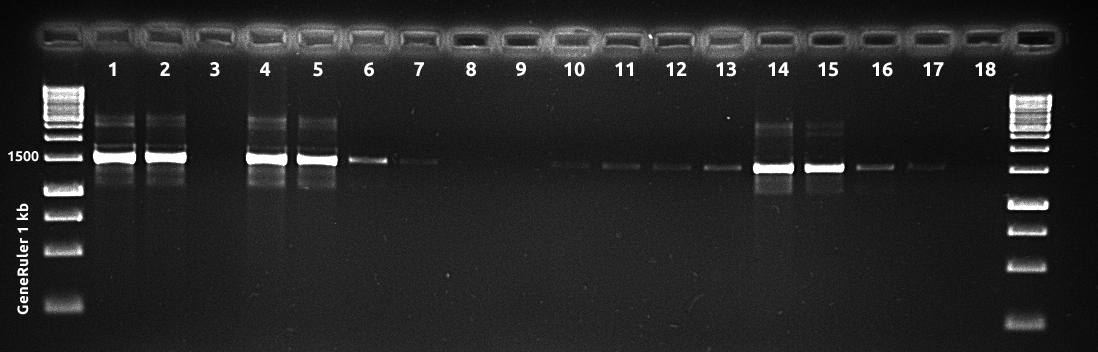

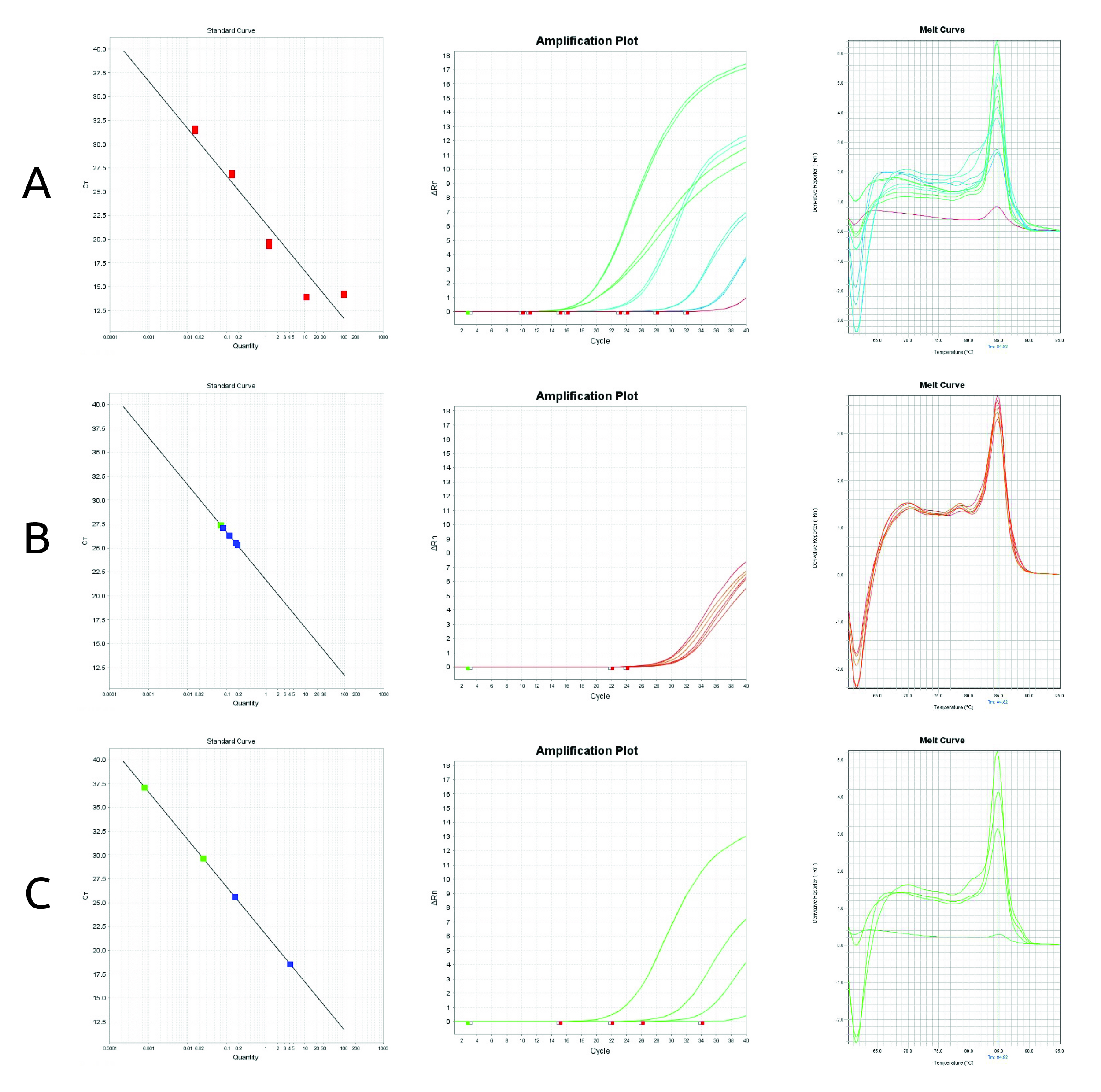

Supplement: Supplementary file 1 — Supplementary file1 (DOCX 27 KB) [file 420_2021_1829_MOESM1_ESM.docx]
